# IVT-SAPAS: Low-Input and Rapid Method for Sequencing Alternative Polyadenylation Sites

**DOI:** 10.1371/journal.pone.0145477

**Published:** 2015-12-28

**Authors:** Yonggui Fu, Yutong Ge, Yu Sun, Jiahui Liang, Liang Wan, Xiaojian Wu, Anlong Xu

**Affiliations:** 1 Shenzhen Research Center of State Key Laboratory for Biocontrol, Research Institute of Sun Yat-sen University at Shenzhen, Shenzhen Virtual University Park, Hi-tech Industrial Park, Nanshan District, Shenzhen, 518057, P. R. China; 2 State Key Laboratory for Biocontrol, Guangdong Province Key Laboratory of Pharmaceutical Functional Genes, Department of Biochemistry, School of Life Sciences, Sun Yat-sen University, Higher Education Mega Center, Guangzhou, 510006, P. R. China; 3 The Sixth Affiliated Hospital, Sun Yat-Sen University, 26 Yuancun Erheng Rd, Guangzhou, Guangdong, 510655, P. R. China; 4 Beijing University of Chinese Medicine, 11 Bei San Huan Dong Road, Chao-yang District, Beijing, 100029, P. R. China; Huazhong University of Science and Technology, CHINA

## Abstract

Gene transcribing with alternative polyadenylation (APA) sites leads to mRNA isoforms, which may encode different proteins or harbor different 3'UTRs. APA plays an important role in regulating gene expression network among various physiological processes, such as development, immune responses and cancer. Several methods of library construction for APA study have been developed to apply high-throughput sequencing. However, the requirement of high-input RNA and time-consuming nature of the current methods limited the studies of APA for the samples difficult to obtain. Here, we describe a new method based on our SAPAS in combining *in vitro* transcription (IVT) and magnetic beads purification. The new IVT-SAPAS provides a rapid and high-parallel procedure for APA library construction with low-input sample, which may be a new robust approach for studying APA.

## Introduction

Transcription termination by RNA Pol II (RNAPII) in eukaryotes is conducted with recognizing the poly(A) signals by cleavage and polyadenylation complex factors. More than half of the genes harbor alternative polyadenylation (APA) sites. Tandem APA sites located in 3'UTR region can lead to transcription of different mRNA isoforms with various 3'UTRs. Various biological effects associated with tandem APA were investigated, including cancer transformation [1, 2], embryonic development [3–5] immune responses [6] and neuronal activity [7]. These studies found that shorter 3’UTRs may contribute to a higher proliferation rate of cells, cancer transformation, and responses to stimuli. APA sites located in upstream of canonical stop codon can produce short mRNA isoforms, resulting in changing the C terminal amino acids sequences of proteins. One of the well known examples is the gene encoding μ chain of IgM molecule, the APA sites of which lead to one membrane-bound and one secreted form of IgM [8–10].

Noticed the important function of APA, several groups [2, 4, 11–14] developed methods of Poly(A) sequencing independently to profile APA in genome wide fashion with the second generation sequencing technology. However, tens of μg total RNA are required to construct poly(A) sites sequencing library [2, 4, 11]. Such higher input samples of these methods will undoubtedly restrict their application in studies with limited amount of total RNAs such as early fetal development, cancer and immune subset cells. Here, based on our method of SAPAS (sequencing alternative polyadenylation sites), we developed a new method called IVT-SAPAS by integrating *in vitro* transcription and magnetic bead-based purification and size selection. With the new method, as low as 200 ng of total RNA is sufficient to obtain 3' end library, benefiting from the good linear amplification of in vitro-transcription and high recovery rate of magnetic bead-based method. At the same time, the magnetic bead-based purification and size selection method also makes the library preparation high-parallel.

## Materials and Methods

### Cell cultures and total RNA extraction

A breast cancer cell line MCF7 (a gift from Dr. Erwei Song’s lab, Department of Breast Surgery, No. 2 Affiliated Hospital, Sun Yat-sen University, Guangzhou, China) was cultured in Dulbecco’s modified Eagle’s medium (DMEM), and a human normal mammary epithelial cell line MCF10A (a gift from Qiang Liu’s lab, State Key Laboratory of Oncology in South China, Sun Yat-sen University, Guangzhou, China) was cultured in monolayer in DMEM/F12. Total RNA was extracted from the cells using QIAGEN RNeasy^®^ Mini kits, and maintained in RNase-free water. The quality of the samples was checked with agarose gel electrophoresis and OD260/280 ratio greater than two.

### IVT-SAPAS library preparation

**1) RNA fragmentation.** About 200 ng total RNA for each sample was randomly fragmented with heating at 80°C for 30 minutes. **2) The first round of reverse transcription.** An anchored oligo d(T) tagged with Illumina A adaptor and T7 promoter was used for the first strand cDNA synthesis, and then the second strand was synthesized with RNase H, E. *coli* DNA polymerase and DNA ligase. The frayed termini of double-stranded cDNA were polished with T4 DNA polymerase. Subsequently, the products were purified with Agencourt RNAClean XP kit (Beckman Coulter). **3) In vitro transcription.** RiboMAX^™^ Large Scale RNA Production System-T7 (Promega) was used to perform in vitro transcription with the manufacturer's manual, and then the template cDNA was removed with RQ1 RNase-Free DNase (Promega). The RNA product was also purified with Agencourt RNAClean XP kit (Beckman Coulter). **4) The second round of reverse transcription.** Random primers tagged with part of Illumina B adaptor were used for the first strand cDNA synthesis, and then the product was purified with MinElute PCR Purification Kit (Qiagen). **5) PCR amplification.** Illumina adaptor B with 6-mer barcode and modified oligo d(T) tagged with Illumina adaptor A were used to perform the PCR amplification. Finally, size selection of 200-500bp fragments of the PCR product was performed with AMPure XP Beads (Beckman Coulter), and then the quality of the library was checked with Agilent 2100 Bioanalyzer. The details of protocol and primers were shown in S1 Text and S3 Table.

### Hiseq 2500 sequencing

Twelve libraries with different barcodes were pooled together and sequenced with Hiseq 2500 with rapid run mode. To overcome the problem of the homogeneous nucleotide T of the first 20 bases, 20 dark cycles were taken and then 55 bp were further sequenced.

### Data analysis

The raw reads were mapped to the human genome (hg19) using Bowtie [15] and internal priming was filtered. Next, poly(A) sites were defined for each sample by clustering the unique mapped reads as previously described [2, 5]. And then the poly(A) sites were merged together across samples.

A dataset of genes was constructed by obtaining the genes with the largest 3’UTR for each stop codon from UCSC known genes. The merged poly(A) sites were mapped to these 3'UTRs of the new dataset, and the poly(A) sites mapped to a single 3’UTR were used for further analysis. The expression levels of poly(A) sites were calculated as the number of raw reads scaled to the sample with the lowest number of reads.

To reduce the variance of 3’UTR length across genes, we standardized the length by designating the longest 3’UTR as 1.0 and calculated the weighted mean of 3’UTR length with multiple APA sites for each gene.

A test of linear trend alternative to independence [16] was used to detect the tandem 3'UTR length difference between samples. In brief: 1) A 2xC table was constructed with the number of reads of tandem APA sites for each gene, with tandem APA sites as columns (from the site with shortest UTR to that with longest) and the two samples as rows; 2) The lengths of tandem UTRs represented the scores for the columns, and 0 and 1 the row scores indicating the samples; 3) A Pearson correlation *r* was calculated using the number of reads in the table as the values and the scores for rows and columns as coordinates; and 4) We calculated the statistic *M* ^2^ = (*n*-1)*r* ^2^, which is an approximated *chi*-square distribution with df = 1 for large samples. P-value was obtained, and the Benjamin-Hochberg FDR was estimated with R software.

## Results

### Development of IVT-SAPAS

**Fig 1:**
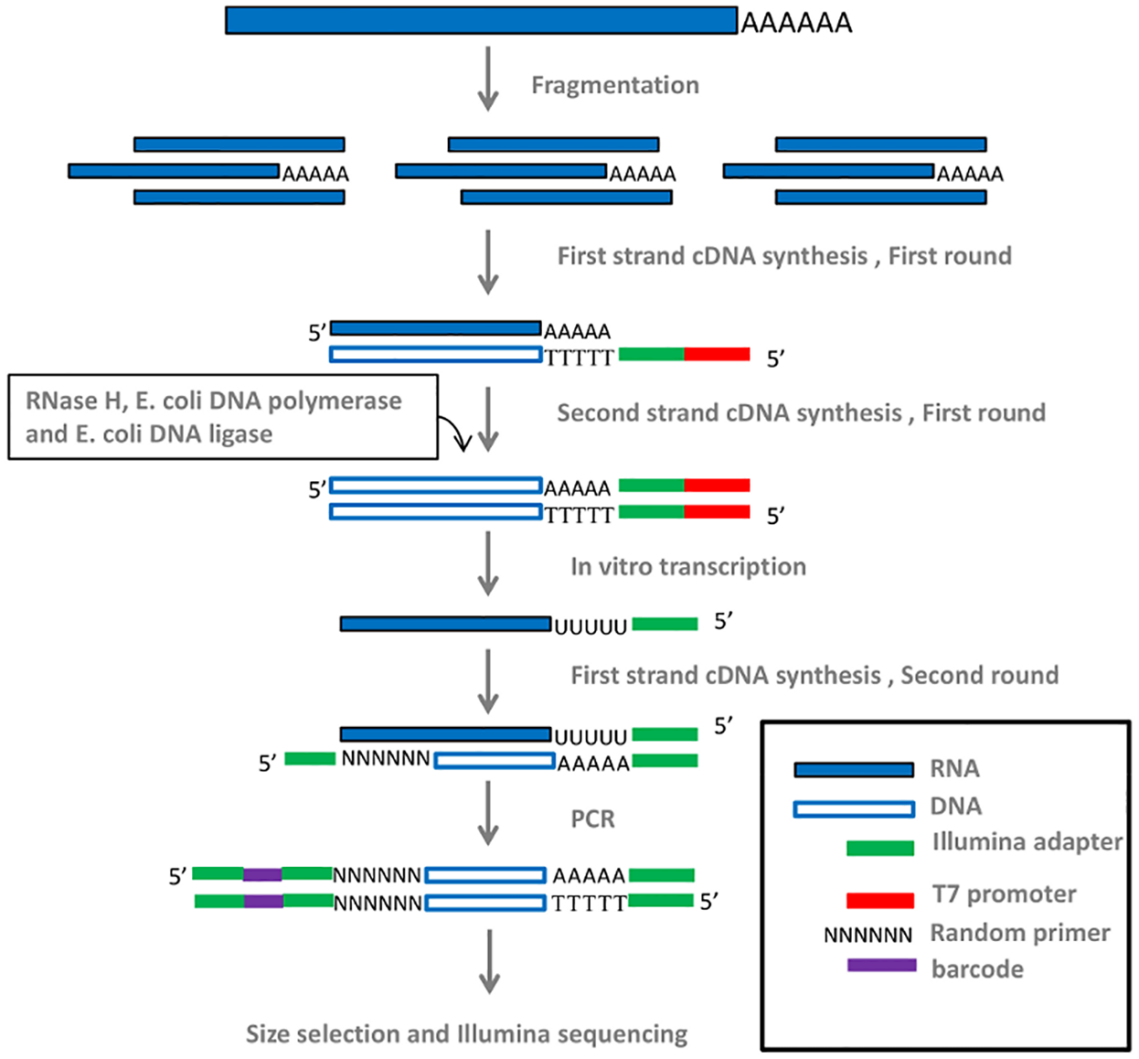
Schematic of IVT-SAPAS method. The method mainly includes the steps of RNA fragmentation, two rounds of reverse transcription, PCR and size selection. See the method part for details.

The current methods of poly(A) sequencing, including our SAPAS method [2], require abundant total RNA, which reduces the success rate of library preparation and limits their usefulness. By integrating SAPAS, *in vitro* transcription with magnetic bead based purification and size selection, we developed IVT-SAPAS (Fig 1). Briefly, after fragmentation of RNA by heating and reverse transcription with anchored oligo d(T), *in vitro* transcription was used to effectively amplify the samples linearly. Subsequently, a second round of reverse transcription and PCR were conducted. Finally, the PCR product of 300–500 bp was selected with AMPure XP beads.

Using IVT-SAPAS, with 200 ng of initial total RNA, we profiled poly(A) sites of a human mammary epithelial cell line (MCF10A) and a breast cancer cell line (MCF7) with Hiseq 2500 rapid mode. Three biological replicates were performed for each cell line. On average, 27.9 million raw reads for each sample were obtained (S1 Table). In total, we obtained 76,698 poly(A) sites with a threshold of 12 reads for the 6 samples, covering 17,145 genes.

**Fig 2:**
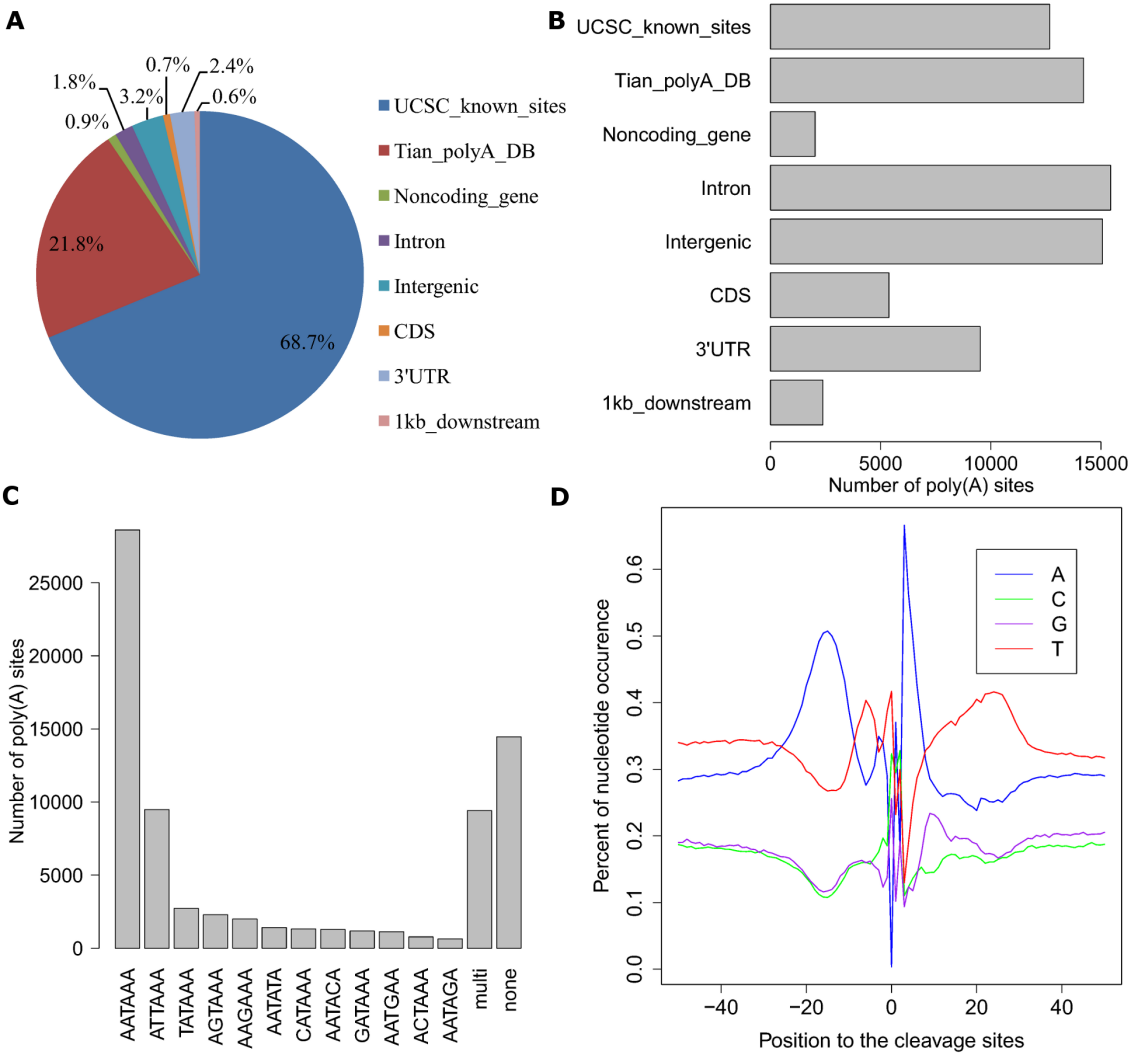
Characteristics of poly(A) sites. The reads and poly(A) sites were firstly mapped to the known sites of UCSC and Tian's poly(A) database, and the unmapped were annotated to 3'UTR, intron, CDS, 1kb_downstream and intergenic region. A) Pie-chart of mappig location of reads; B) Distribution of mapping location of poly(A) sites; C) Distribution of poly(A) signals; D) Nucleotide composition flanked poly(A) sites.

To check the poly(A) site sequencing efficacy of IVT-SAPAS, we first annotated the reads and poly(A) sites to known poly(A) sites, 3'UTRs, introns, CDS, 1 kb downstream, and noncoding gene and intergenic regions. We found that 90.5% of reads could be mapped to poly(A) sites in the UCSC and Tian's poly(A) databases (Fig 2A), and 35% of poly(A) sites were mapped to known poly(A) sites (Fig 2B). This was consistent with our previous SAPAS results [2]. We also found distribution of poly(A) signals to be similar among poly(A) categories (Fig 2C). The strongest AATAAA and ATTAAA signals were common to all poly(A) categories except CDS. Transcriptional termination and cleavage factors mainly recognized the region of -50 to +50 bp of a poly(A) site. Nucleotide composition of all poly(A) sites showed an A-rich region from positions -25 to -10 and a T-rich region at approximately position +20 (Fig 2D). These two regions correspond to the poly(A) signal and binding sites for CstF, an important member of the 3’ end processing complex [17]. All categories of poly(A) sites except CDS showed this pattern (S1 Fig), suggesting the validity of IVT-SAPAS in capturing poly(A) sites.

Some genes have multiple stop condons by the interaction of alternative splicing and APA, then we constructed a gene dataset by selecting the known genes with the largest 3’UTR for each stop codon from UCSC known genes database. We mapped the poly(A) sites to these 3'UTRs of the new dataset. In total, 10,341 genes were mapped with 9,812 UCSC known gene clusters. We created a scatter plot and calculated pairwise correlation coefficients (R^2^) of poly(A) site expression levels (S2 Fig). The average correlation coefficients among replicate samples of MCF7 and MCF10A were 0.95 and 0.92, respectively, suggesting good reproducibility of IVT-SAPAS.

### Shorter 3'UTR in MCF7 than MCF10A

**Fig 3:**
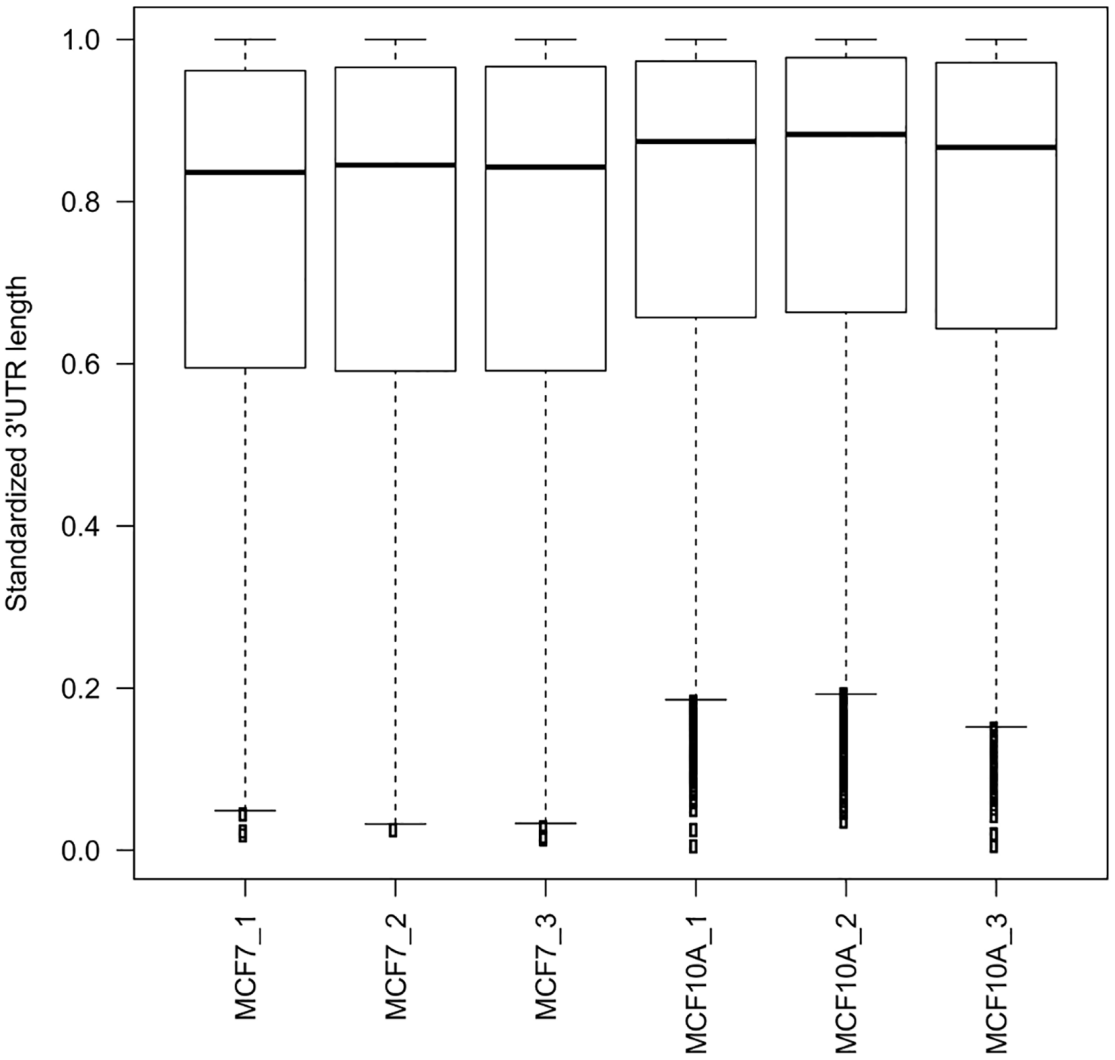
Boxplot of standardized 3'UTR length of genes with tandem APA sites. To reduce the variance of 3’UTR length across genes, we standardized the length by designating the longest 3’UTR as 1.0 and calculated the weighted mean of 3’UTR length with multiple APA sites for each gene.

The 3'UTRs with multiple poly(A) sites were defined as tandem 3'UTRs. These poly(A) sites were tandem APA sites. The data were used for the subsequent analysis. We first calculated the mean standardized 3'UTR length of each gene in each sample. Both box (Fig 3) and scatter plot (S3 Fig) show overall shorter 3'UTR in MCF7 than MCF10A. We then assessed 3'UTR length differences between any pair of MCF7 and MCF10A for each gene with the test of linear trend alternative to independence. In MCF7, a mean of 860 and 137 genes with shortened and lengthened 3'UTRs were found respectively. The number of overlapped genes of the shortened 3'UTRs was 342 and for the lengthened 3'UTRs was 11. Functional annotation with DAVID (http://david.abcc.ncifcrf.gov/) revealed that mitotic cell-cycle-related genes were enriched in genes with shorter 3'UTRs in MCF7 (Enrichment rate = 3.0, FDR = 0.034), including *CCND1* and *CKD6*, which is consistent with our previous study using SAPAS [2].

### APA switching with truncated protein between MCF7 and MCF10A

We found 511 UCSC known gene clusters with different stop codons with reads mapped to 3'UTR regions, which can lead to truncated protein. We summed the poly(A) reads of each gene for each sample as their expression levels. Then, with the mean expression level of each cell line, we compared the gene expression between MCF7 and MCF10A and found 72 significant gene clusters (Fisher Exact test, p≤0.05 corrected by Bonferroni method) (S2 Table). Among them, 57 genes cluster prefer to proximal stop codon, and 15 genes clusters prefer to distal stop codon in MCF7. To validate the results, we performed quantitative RT-PCR for 58 gene clusters. Among them, 50 gene clusters were confirmed (S2 Table).

**Fig 4:**
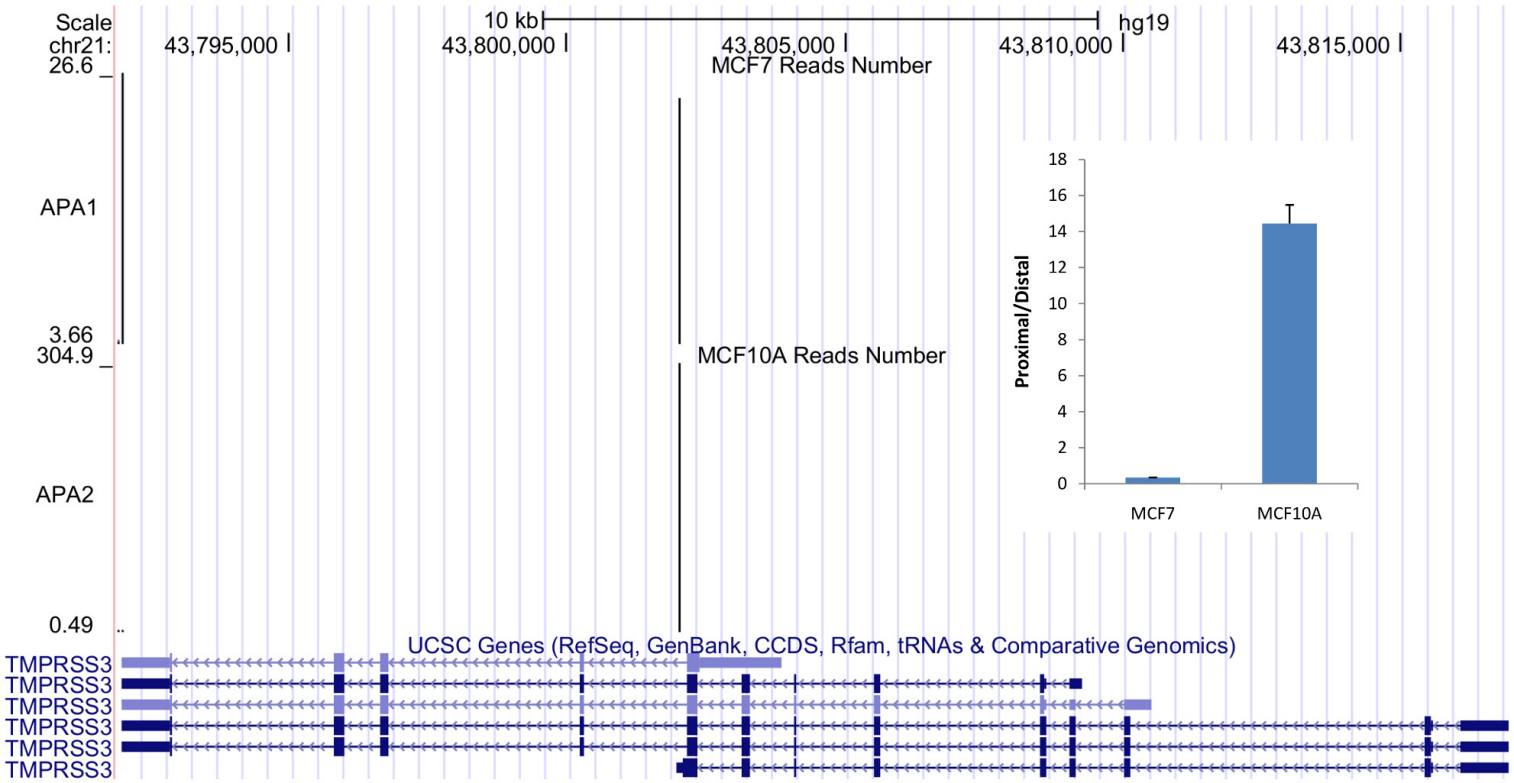
Comparison of TMPRSS3 poly(A) reads distribution between MCF7 and MCF10A. MCF7 prefers to use the full length transcript compared to MCF10A. The inner graph shows qRT-PCR validation (p<0.01 with t test). Two pair of primers (proximal and distal) were used to measure the expression level of the mRNA isoforms.

One of the examples switched to distal stop codon in MCF7 is *TMPRSS3* (Fig 4), encoding a member of the type II trans-membrane serine protease gene family. Several transcript variants are produced by alternative transcription initiation, alternative splicing and alternative polyadenylation. The full length protein encoded by transcript variant A contains a serine protease domain, a trans-membrane domain, an LDL receptor-like domain, and a scavenger receptor cysteine-rich domain. The truncated TMPRSS3-D variant lacks part of protease domain. *TMPRSS3-A* transcript was found to have elevated expression in pancreatic, gastric, colorectal cancer [18]. Over-expression of *TMPRSS3-D* transcript was also found in ovarian carcinomas [19]. Here we found elevated expression of *TMPRSS3-A* and reduced expression of *TMPRSS3-D* transcript in MCF7 compared to MCF10A with IVT-SAPAS. And qRT-PCR was also confirmed the switching. This result suggests that alternative transcript with coding region changes due to APA may play an important role in cancer development.

## Discussion

Here, based on SAPAS method, we developed a new APA sequencing method by integrating *in vitro* transcription and magnetic beads-based on purification and size selection. This new IVT-SAPAS method has advantages of lower-input samples, and high-parallel and time-saving.

At least 1 μg of total RNA is usually needed to prepare RNA-seq library with Illumina standard protocol. The average length of human mRNA is about 2 kb and only 200bp upstream of poly(A) are usually amplified for poly(A) sites sequencing, then more abundant total RNA are usually required for these methods than RNA-seq. Actually tens of μg total RNA are required for previous methods to construct poly(A) sites sequencing library [2, 4, 11, 14]. The *in vitro* transcription of IVT-SAPAS method reduced the input of total RNA to 200ng, which could be used for APA research for the samples difficult to obtain. In the previous SAPAS method, we purified cDNA with centrifuge columns and performed size selection with PAGE. Here, we used Agencourt RNAclean XP magnetic beads and Ampure XP magnetic beads for purification and size selection, which made IVT-SAPAS method high-parallel and time-saving.

In this method, we directly add the Illumina adaptors by reverse transcription and PCR, which reduces the difficulty of the experiment comparing to 3P-seq [11] and 3'READS [12]. However, direct sequencing this IVT-SAPAS library will encounter the homopolymer problem introduced by oligo d(T) primer. Here we successfully overcame this problem by performing 20 dark cycles (chemistry-only cycles) firstly.

We found genome wide shorter 3'UTRs in cancer cell than normal cell, which is consistent with previous findings [1, 2]. Furthermore, for the APA sites that impact the coding region sequences, we also found the cancer cell preferred to use promoter-proximal poly(A) sites. This global trend of shorter transcripts in cancer cell is consistent with the finding on embryonic development and cell differentiation [12].

## Supporting Information

S1 Fig Nucleotide profile flanking poly(A) sites. The poly(A) sites were classified into eight classes as described in text.
(PDF)

S2 Fig Scatter plot of expression levels of poly(A) sites. (PDF)

S3 Fig Scatter plot of standardized 3'UTR length in MCF7 and MCF10A. The red lines show the diagonal lines.
(PDF)

S1 Table Summary statistics of IVT-SAPAS data. (DOCX)

S2 Table Genes with APA switching between MCF7 and MCF10A which lead to coding region changes. p values were corrected by bonferroni method. The genes confirmed by qRT-PCR were labeled by yellow color.
(DOCX)

S3 Table Primers used in IVT-SAPAS experiment. (DOCX)

S1 Text The protocol of IVT-SAPAS. (DOCX)
