# Supplementary figures and images for: IVT-SAPAS: Low-Input and Rapid Method for Sequencing Alternative Polyadenylation Sites
Source: PLoS One. 2015 Dec 28;10(12):e0145477. doi: 10.1371/journal.pone.0145477 (PMC4692544; doi:10.1371/journal.pone.0145477)

UCSC

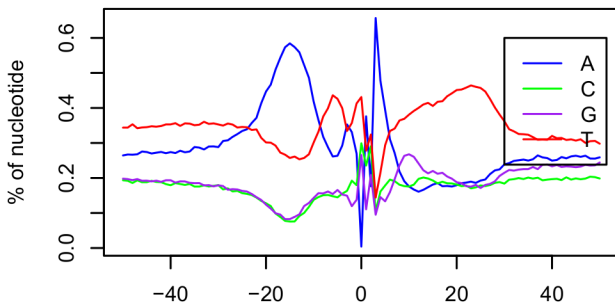

Tian\_poly(A)\_DB

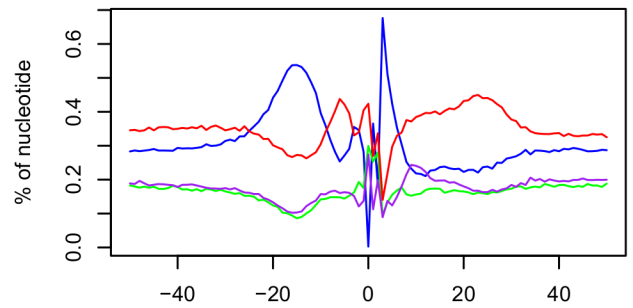

Noncoding\_gene

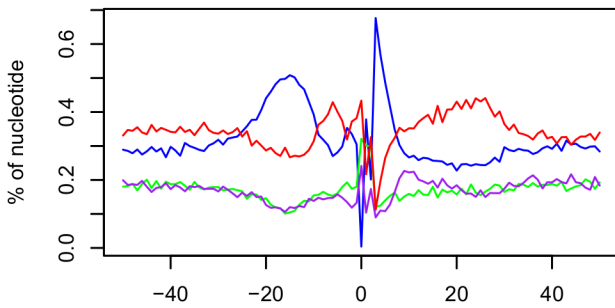

Intron

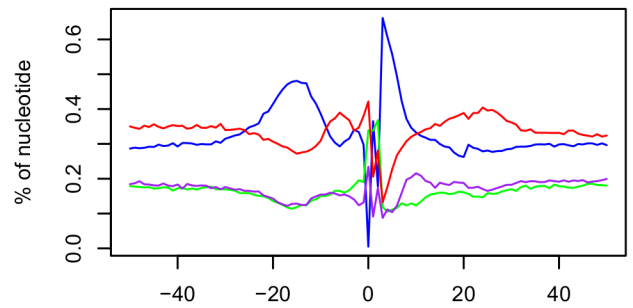

Intergenic

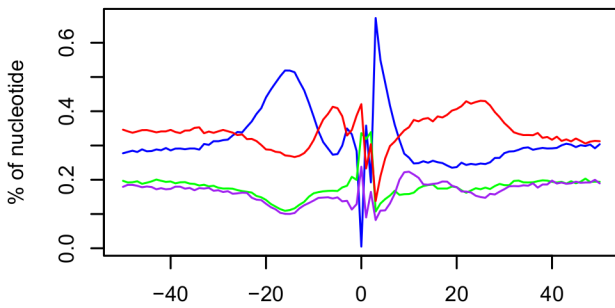

CDS

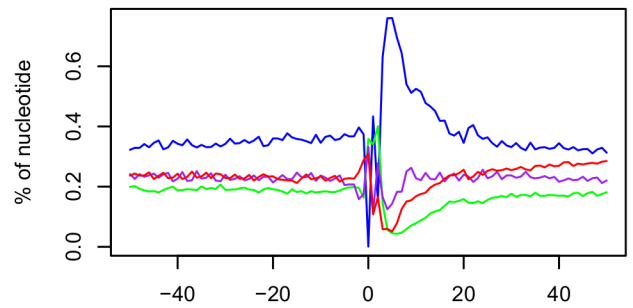

3'UTR

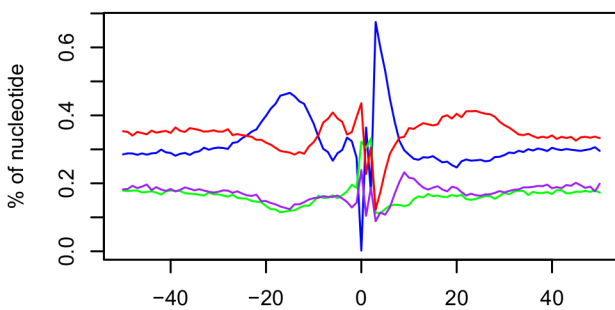

1kb\_dwonstream

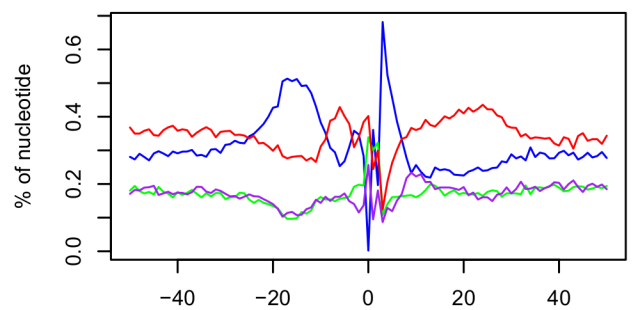

Supplement: S1 Fig — The poly(A) sites were classified into eight classes as described in text. (PDF) [file pone.0145477.s001.pdf]

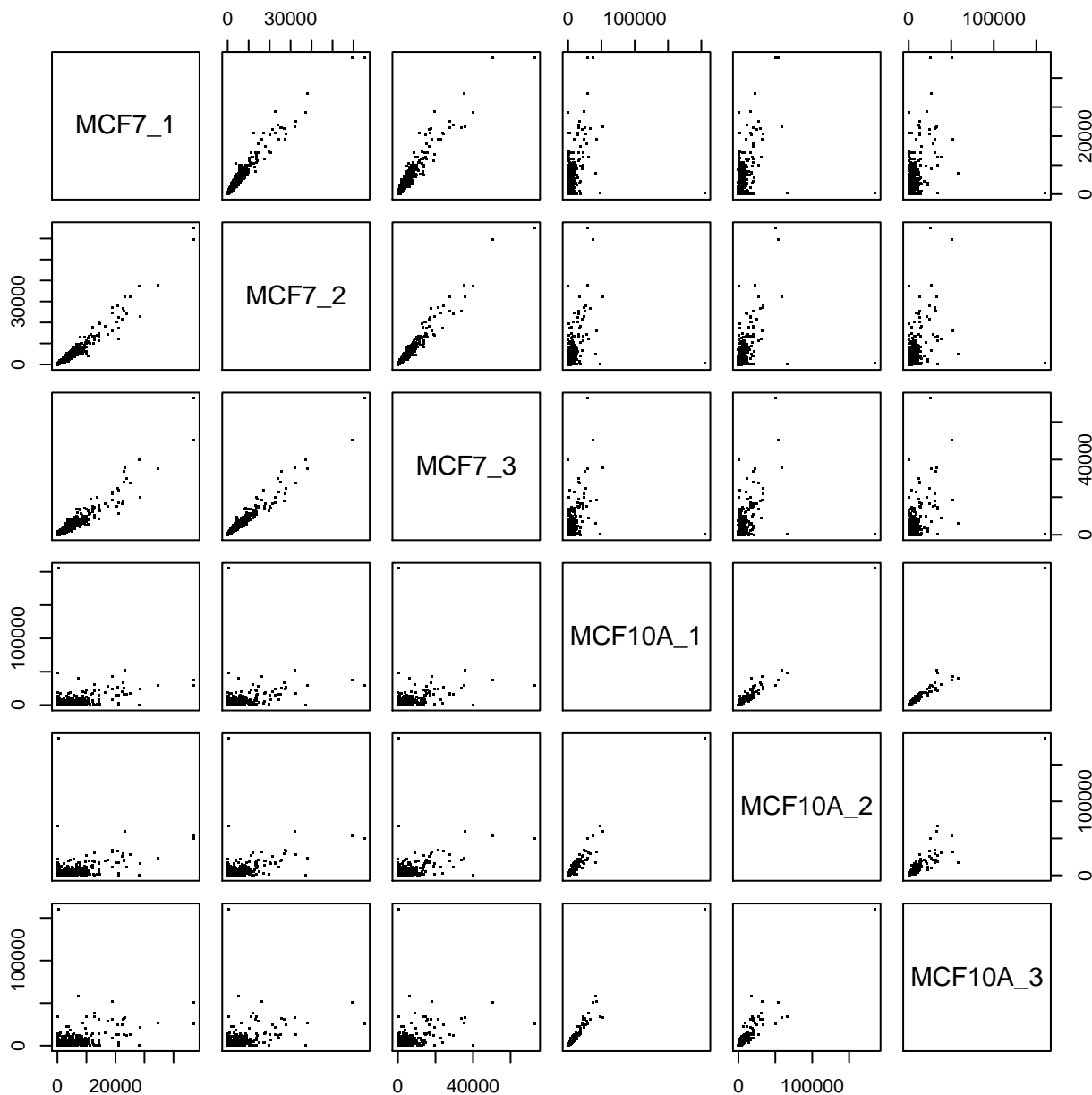

Supplement: S2 Fig — (PDF) [file pone.0145477.s002.pdf]

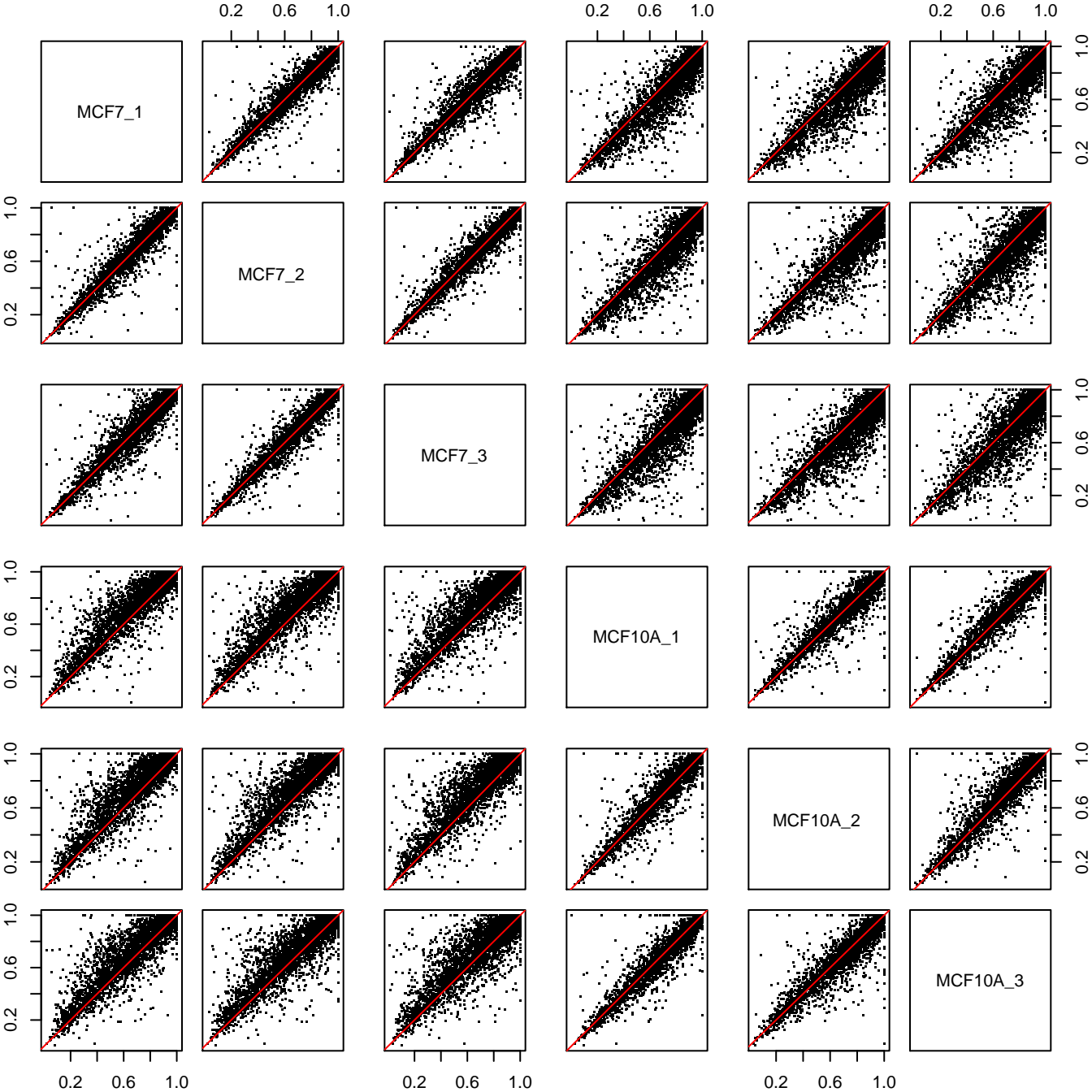

Supplement: S3 Fig — The red lines show the diagonal lines. (PDF) [file pone.0145477.s003.pdf]
