# Supplementary material for: IVT-SAPAS: Low-Input and Rapid Method for Sequencing Alternative Polyadenylation Sites
Source: PLoS One. 2015 Dec 28;10(12):e0145477. doi: 10.1371/journal.pone.0145477 (PMC4692544; doi:10.1371/journal.pone.0145477)
Supplement: S1 Table — (DOCX) [file pone.0145477.s004.docx]

S1 Table. **Summary statistics of IVT-SAPAS data**

| Samples | MCF7_1 | MCF7_2 | MCF7_3 | MCF10A_1 | MCF10A_2 | MCF10A_3 | Total |
| --- | --- | --- | --- | --- | --- | --- | --- |
| Raw reads | 25,505,454 | 28,079,882 | 24,349,281 | 28,978,498 | 29,114,192 | 31,299,927 | 167,327,234 |
| Qualified reads | 25,406,947 | 27,881,155 | 24,224,784 | 28,890,948 | 29,018,325 | 31,299,737 | 166,721,896 (99.6%) |
| Mapped to genome | 20,431,870 | 23,708,260 | 21,147,393 | 25,391,323 | 25,753,460 | 24,909,140 | 141,341,446 (84.5%) |
| Uniquely mapped to genome | 13,660,185 | 15,953,308 | 14,052,006 | 16,407,378 | 17,350,216 | 15,714,382 | 93,137,475 (55.7%) |
| Mapped to nuclear genome | 10,149,535 | 11,960,953 | 9,760,329 | 12,665,226 | 13,635,537 | 12,356,747 | 70,528,327(42.1%) |
| After IP filter | 8,451,925 | 10,811,136 | 9,001,379 | 10,797,053 | 12,257,935 | 9,955,030 | 61,274,458(36.6%) |
| Genes sampled by reads |  |  |  |  |  |  | 18,748 |
| All poly(A) sites |  |  |  |  |  |  | 76,698 |
| Poly(A) sites mapped to 3'UTR region |  |  |  |  |  |  | 23,082 |
