# Supplementary material for: IVT-SAPAS: Low-Input and Rapid Method for Sequencing Alternative Polyadenylation Sites
Source: PLoS One. 2015 Dec 28;10(12):e0145477. doi: 10.1371/journal.pone.0145477 (PMC4692544; doi:10.1371/journal.pone.0145477)
Supplement: S2 Table — p values were corrected by bonferroni method. The genes confirmed by qRT-PCR were labeled by yellow color. (DOCX) [file pone.0145477.s005.docx]

**S2 Table. Genes with APA switching between MCF7 and MCF10A which lead to coding region changes.** p values were corrected by bonferroni method. The genes confirmed by qRT-PCR were labeled by yellow color.

| Gene Symbol | UCSC isoform | | IVT_SAPAS reads number | | | | P value | (MCF7_P/MCF7_D)/(MCF10A_P/MCF10A_D) | |
| --- | --- | --- | --- | --- | --- | --- | --- | --- | --- |
|  | Proximal(P) | Distal(D) | MCF7_P | MCF10A_P | MCF7_D | MCF10A_D |  | Sequencing | qRT-PCR |
| SSU72 | uc001age.1 | uc001agd.2 | 202.84333 | 54.505 | 1529.551 | 1921.503 | 9.73E-25 | 4.68 | 3.60 |
| HP1BP3 | uc001bea.2 | uc010odf.1 | 840.583 | 421.718 | 62.736 | 67.417 | 3.37E-02 | 2.14 | 0.25 |
| USP48 | uc001bff.2 | uc001bfa.2 | 82.727333 | 51.10367 | 167.0407 | 347.574 | 3.78E-07 | 3.37 | 1.53 |
| ITGB3BP | uc001dbe.1 | uc001dba.1 | 16.230667 | 82.50367 | 280.197 | 375.7933 | 7.45E-05 | 0.26 |  |
| CD58 | uc001egp.3 | uc001egm.2 | 362.67067 | 37.421 | 86.16967 | 56.78 | 2.79E-12 | 6.39 |  |
| SLC45A3 | uc001hda.1 | uc001hcy.1 | 27.992 | 86.086 | 34.10233 | 4.692 | 2.25E-09 | 0.04 |  |
| MTPAP | uc001ivc.2 | uc001iva.3 | 26.227667 | 75.167 | 383.386 | 327.568 | 5.84E-05 | 0.30 | 1.66 |
| NRP1 | uc001ixc.1 | uc001iwv.3 | 25.165667 | 31.443 | 630.4043 | 216.1053 | 3.59E-03 | 0.27 | 1.04 |
| HERC4 | uc001jnk.2 | uc009xpq.2 | 16.365667 | 7.142 | 679.1673 | 2145.851 | 2.91E-03 | 7.24 |  |
| CHST15 | uc010que.1 | uc001lhl.2 | 25.856333 | 4.798333 | 402.4783 | 514.0727 | 5.02E-03 | 6.88 | 0.13 |
| FBXO3 | uc001mva.1 | uc010rej.1 | 103.76267 | 66.96067 | 105.1313 | 155.589 | 1.74E-02 | 2.29 | 1.79 |
| AHNAK | uc001ntl.2 | uc001ntk.1 | 4473.851 | 8176.962 | 63.971 | 228.7573 | 4.34E-04 | 1.96 |  |
| MPZL2 | uc001psp.1 | uc001psn.2 | 42.380667 | 7.605667 | 365.4087 | 551.6267 | 4.67E-07 | 8.41 | 1.40 |
| CDCA3 | uc001qrg.2 | uc001qre.2 | 303.90833 | 346.9327 | 57.64367 | 24.214 | 2.79E-02 | 0.37 | 1.20 |
| TUBA1A | uc009zlf.2 | uc001rtk.2 | 458.22967 | 123.979 | 35942.95 | 26225.27 | 2.66E-23 | 2.70 |  |
| GALNT4 | uc001tbd.2 | uc001tba.2 | 410.22333 | 129.5317 | 327.519 | 187.334 | 8.46E-03 | 1.81 | 6.12 |
| ANAPC7 | uc001tqp.3 | uc001tqo.2 | 413.58467 | 181.798 | 651.6543 | 498.6973 | 8.55E-05 | 1.74 | 1.65 |
| ANKLE2 | uc001uky.3 | uc009zyw.1 | 28.648667 | 5.135 | 276.5073 | 446.3193 | 3.13E-05 | 9.01 | 2.16 |
| INTS6 | uc001vfm.2 | uc001vfi.2 | 135.84367 | 10.41033 | 594.7967 | 789.5703 | 3.11E-32 | 17.32 | 12.55 |
| EFCAB11 | uc001xxw.1 | uc001xxs.2 | 172.681 | 9.358333 | 37.50867 | 22.669 | 1.05E-06 | 11.15 | 13.61 |
| RFX7 | uc010bfn.2 | uc002adn.1 | 107.57867 | 279.339 | 35.08133 | 243.851 | 7.44E-04 | 2.68 |  |
| ZNF280D | uc002adx.2 | uc002adt.2 | 49.109333 | 18.426 | 26.99467 | 75.43333 | 1.31E-06 | 7.45 | 5.70 |
| C15orf38 | uc002bou.2 | uc002boq.3 | 115.98267 | 13.863 | 554.2433 | 332.8563 | 8.44E-08 | 5.02 | 0.87 |
| PLEKHM1 | uc002ijc.2 | uc002ija.2 | 47.857333 | 24.346 | 119.23 | 177.1167 | 3.32E-02 | 2.92 | 1.53 |
| CCDC47 | uc002jbt.2 | uc002jbs.3 | 0.3333333 | 25.70833 | 991.6383 | 290.646 | 2.22E-14 | 0.00 | 1.15 |
| RPRD1A | uc010dmx.2 | uc002kzg.2 | 167.33033 | 27.45667 | 212.9437 | 178.9537 | 2.08E-12 | 5.12 |  |
| SLC39A6 | uc002kzj.2 | uc010dmy.2 | 54.528333 | 1.287 | 3980.325 | 1008.022 | 3.87E-02 | 10.73 |  |
| TPGS2 | uc002kzw.1 | uc002kzv.1 | 1975.0313 | 1033.774 | 36.98767 | 77.46933 | 1.02E-09 | 4.00 | 1.04 |
| TPGS2 | uc002kzy.3 | uc002kzv.1 | 17.545 | 4.475667 | 36.98767 | 77.46933 | 1.21E-02 | 8.21 |  |
| SMAD2 | uc010xdd.1 | uc002lcy.2 | 34.495 | 7.450667 | 422.2017 | 421.707 | 1.86E-02 | 4.62 | 1.46 |
| PTPRS | uc002mbz.1 | uc010xin.1 | 0 | 10.80767 | 196.659 | 76.29833 | 7.17E-04 | 0.00 | 0.08 |
| EPS15L1 | uc002neb.1 | uc002ndx.2 | 602.35133 | 63.32333 | 117.129 | 40.82067 | 1.07E-04 | 3.32 | 1.62 |
| OPA3 | uc002pck.3 | uc002pcj.3 | 84.32 | 80.852 | 98.89533 | 9.272667 | 1.26E-10 | 0.10 | 0.54 |
| NOL10 | uc002rar.2 | uc002raq.2 | 40.626 | 3.594333 | 490.6447 | 382.1177 | 6.39E-04 | 8.80 | 1.39 |
| GCFC2 | uc002snp.3 | uc010ffs.2 | 63.822333 | 94.24567 | 13.97 | 84.96767 | 3.26E-03 | 4.12 | 2.71 |
| FHL2 | uc002tde.1 | uc002tct.2 | 10.301 | 5.165333 | 253.925 | 1399.619 | 6.16E-03 | 10.99 | 2.71 |
| WDR33 | uc002tpi.1 | uc002tpg.1 | 765.83667 | 384.483 | 124.1 | 188.4487 | 1.10E-14 | 3.02 | 1.19 |
| STAT1 | uc002usk.2 | uc002usj.2 | 1670.811 | 280.206 | 7765.395 | 954.366 | 1.65E-02 | 0.73 | 0.60 |
| ALS2 | uc002uyp.3 | uc002uyo.2 | 28.66 | 17.21667 | 614.6147 | 1315.007 | 1.05E-02 | 3.56 | 1.99 |
| ALS2 | uc002uyr.2 | uc002uyo.2 | 84.517 | 17.619 | 614.6147 | 1315.007 | 4.13E-22 | 10.26 | 7.24 |
| KIF16B | uc010gci.1 | uc002wpe.1 | 120.354 | 43.13933 | 90.754 | 127.4787 | 2.89E-07 | 3.92 | 1.68 |
| SNX5 | uc010zrt.1 | uc002wqc.2 | 192.98133 | 119.6553 | 1948.855 | 2042.467 | 6.67E-03 | 1.69 | 1.04 |
| ABHD12 | uc002wus.1 | uc002wuq.2 | 1082.0567 | 1123.775 | 114.5553 | 28.40067 | 4.24E-11 | 0.24 | 0.28 |
| C20orf112 | uc002wxv.3 | uc002wxu.3 | 45.658333 | 83.23533 | 1059.346 | 848.426 | 7.49E-03 | 0.44 | 0.96 |
| UQCC | uc002xci.1 | uc002xcc.2 | 91.924333 | 121.791 | 182.1087 | 674.0763 | 2.47E-07 | 2.79 | 3.74 |
| TMPRSS3 | uc002zbd.2 | uc002zay.2 | 24.361333 | 304.9017 | 30.25733 | 3.197667 | 4.97E-23 | 0.01 | 0.02 |
| C22orf39 | uc002zpk.1 | uc002zpi.2 | 187.98733 | 49.72033 | 183.4433 | 172.4457 | 4.81E-09 | 3.55 | 3.83 |
| RPUSD3 | uc003btn.2 | uc011atk.1 | 53.696333 | 84.471 | 2291.379 | 1751.644 | 2.73E-02 | 0.49 | 0.49 |
| SEC22C | uc003clj.2 | uc003clh.2 | 37.388 | 22.277 | 73.775 | 178.8687 | 1.79E-03 | 4.07 | 1.18 |
| PDS5A | uc003guw.3 | uc010ifo.2 | 119.93833 | 81.191 | 941.5943 | 1399.862 | 7.03E-05 | 2.20 | 2.05 |
| HNRNPD | uc010ijr.1 | uc003hmm.1 | 36.488333 | 24.664 | 67.86467 | 622.884 | 1.06E-15 | 13.58 |  |
| HELQ | uc003hoo.1 | uc003hom.2 | 41.887667 | 20.994 | 73.11367 | 148.569 | 1.14E-03 | 4.05 |  |
| SCLT1 | uc003igr.2 | uc003ign.2 | 77.981667 | 13.42167 | 11.21867 | 33.04867 | 2.68E-09 | 17.12 | 8.73 |
| SCLT1 | uc003igt.3 | uc003ign.2 | 19.381 | 5.854667 | 11.21867 | 33.04867 | 2.63E-02 | 9.75 | 2.91 |
| MYO10 | uc003jfv.2 | uc011cnb.1 | 27.189333 | 5.412667 | 80.09467 | 194.8177 | 7.95E-07 | 12.22 | 4.18 |
| TMED7 | uc011cwd.1 | uc003krc.2 | 363.14633 | 230.1223 | 20.16267 | 59.20867 | 1.05E-06 | 4.63 | 3.15 |
| DST | uc003pdc.3 | uc003pcv.3 | 20.452 | 597.051 | 72.37633 | 157.297 | 5.06E-25 | 0.07 | 0.10 |
| SYNCRIP | uc003pkz.2 | uc003pku.2 | 449.48433 | 424.843 | 1143.807 | 2027.06 | 2.35E-13 | 1.87 | 3.50 |
| USP45 | uc003ppz.2 | uc003ppw.2 | 200.39 | 142.8493 | 30.41333 | 85.54433 | 5.78E-07 | 3.95 |  |
| SOD2 | uc003qsi.1 | uc003qsg.2 | 234.84733 | 112.3097 | 22.86 | 36.87033 | 1.07E-02 | 3.37 |  |
| PHF10 | uc011eha.1 | uc011egy.1 | 164.02 | 112.652 | 456.9243 | 604.721 | 1.02E-03 | 1.93 | 2.25 |
| ZC3HAV1 | uc003vuo.2 | uc003vun.2 | 186.62433 | 60.19 | 44.731 | 46.18867 | 5.00E-03 | 3.20 | 3.96 |
| NCAPG2 | uc011kwd.1 | uc011kwc.1 | 177.81433 | 11.65567 | 534.2613 | 278.4497 | 1.97E-14 | 7.95 | 3.09 |
| CNOT7 | uc003wxh.1 | uc003wxf.1 | 62.316333 | 25.001 | 692.474 | 793.3027 | 3.84E-03 | 2.86 | 1.63 |
| TMEM68 | uc003xsi.1 | uc003xsg.1 | 53.949333 | 45.44933 | 243.2673 | 498.0047 | 2.32E-02 | 2.43 | 3.95 |
| KIAA1429 | uc003ygp.2 | uc003ygo.1 | 132.118 | 30.659 | 127.5193 | 90.66 | 1.41E-03 | 3.06 | 5.21 |
| CYHR1 | uc003zcw.2 | uc003zcv.2 | 164.25833 | 49.00367 | 24.631 | 28.972 | 1.10E-02 | 3.94 | 6.09 |
| PLAA | uc003zqe.2 | uc003zqd.2 | 441.722 | 231.6733 | 131.716 | 186.3017 | 5.02E-10 | 2.70 |  |
| MAPKAP1 | uc004bqa.2 | uc011lzt.1 | 198.60767 | 42.292 | 823.1783 | 1354.323 | 7.12E-39 | 7.73 | 12.04 |
| FNBP1 | uc004byx.1 | uc011mbu.1 | 323.608 | 67.50867 | 319.3953 | 134.3603 | 1.78E-02 | 2.02 | 1.56 |
| HDAC8 | uc004eav.2 | uc011mqe.1 | 38.745667 | 15.64367 | 12.44833 | 44.86667 | 8.56E-05 | 8.93 | 4.87 |
| LAMP2 | uc011mua.1 | uc004est.3 | 2228.8803 | 2014.89 | 1228.841 | 389.904 | 2.40E-59 | 0.35 | 2.59 |
