# Supplementary material for: IVT-SAPAS: Low-Input and Rapid Method for Sequencing Alternative Polyadenylation Sites
Source: PLoS One. 2015 Dec 28;10(12):e0145477. doi: 10.1371/journal.pone.0145477 (PMC4692544; doi:10.1371/journal.pone.0145477)
Supplement: S3 Table — (DOCX) [file pone.0145477.s006.docx]

Table S3. Primers used in IVT-SAPAS experiment.

| Primer | Usage | Sequence |
| --- | --- | --- |
| T7-IlluminaA-oligod(T) | First round RT | AAGCTTAGATATCTAATACGACTCACTATAGGGACCTACACGACGCTCTTCCGATCTTTTTTTTTTTTTTTTTTTTV |
| IlluminaB-random-primer | Second round RT | AGTTCAGACGTGCTCTTCCGATCTNNNNNN |
| PrimerA | PCR | AATGATACGGCGACCACCGAGATCTACACTCTTTCCCTACACGACGCTCTTCCGATCTTTTTTcTTTTTTcTTTTTTVN |
| Barcode-PrimerB1 |  | CAAGCAGAAGACGGCATACGAGATCGTGATGTGACTGGAGTTCAGACGTGCTCTTCCGATCT |
| Barcode-PrimerB2 |  | CAAGCAGAAGACGGCATACGAGATACATCGGTGACTGGAGTTCAGACGTGCTCTTCCGATCT |
| Barcode-PrimerB3 |  | CAAGCAGAAGACGGCATACGAGATGCCTAAGTGACTGGAGTTCAGACGTGCTCTTCCGATCT |
| Barcode-PrimerB4 |  | CAAGCAGAAGACGGCATACGAGATTGGTCAGTGACTGGAGTTCAGACGTGCTCTTCCGATCT |
| Barcode-PrimerB5 |  | CAAGCAGAAGACGGCATACGAGATCACTGTGTGACTGGAGTTCAGACGTGCTCTTCCGATCT |
| Barcode-PrimerB6 |  | CAAGCAGAAGACGGCATACGAGATATTGGCGTGACTGGAGTTCAGACGTGCTCTTCCGATCT |
