# Supplementary material for: IVT-SAPAS: Low-Input and Rapid Method for Sequencing Alternative Polyadenylation Sites
Source: PLoS One. 2015 Dec 28;10(12):e0145477. doi: 10.1371/journal.pone.0145477 (PMC4692544; doi:10.1371/journal.pone.0145477)
Supplement: S1 Text — (DOCX) [file pone.0145477.s007.docx]

**IVT-SAPAS experiment protocol** (All of the primers used in the protocol were shown in S3)

**RNA extraction and fragmentation**

About 1×10^6^ cells were used for total RNA extraction by Invitrogen Trizol^®^Reagent and DNA digestion by Ambion TURBO DNA-free^TM^ Kit. Heating without fragmentation buffer was proved to be a useful and convenient method for avoidance of clean-up steps, so we fragmentize about 200ng total RNA for each sample with heating at 94℃ for 20min.

**First reverse- transcription cDNA synthesis**

1. Prepare the following reagents in an autoclaved nuclease–free tube on ice, mix thoroughly and briefly microcentrifugate. Incubate it at 65℃ for 5 minutes, then put it on ice.

| **Reagents** | **Volume(μl)** |
| --- | --- |
| T7-IlluminaA-oligod(T)(1ug/ul) | 1 |
| RNase inhibitor | 0.2 |
| Fragmented total RNA(~500ng) | Up to 10.6 |

1. Prepare the following reagents in an autoclaved nuclease–free tube on ice.

| **Reagents** | **Volume(μl)** |
| --- | --- |
| 5×First strand buffer (Invitrogen) | 4 |
| 0.1M DTT (Invitrogen) | 2 |
| dNTP (10mM,Invitrogen) | 1 |
| RNase inhibitor | 0.4 |
| T4 gene 32 (10mg/ml, NEB) | 1 |
| SuperScriptIII (Invitrogen) | 1 |

1. Mix the contents of the two above tubes by gently pipetting several times. Incubate the samples for 20 thermocycles of 50℃for 2 minutes and 55℃ for 2 minutes.

**Double-strand DNA synthesis and purification**(M-MLV RTase cDNA synthesis kit,TaKaRa)

1. Prepare the following reagents in an autoclaved nuclease–free tube on ice, mix thoroughly and briefly microcentrifugate.

| **Reagents** | **Volume(μl)** |
| --- | --- |
| Above first strand reaction solution | 20 |
| 5×Second strand reaction solution | 15 |
| dNTP (10mM) | 1.5 |
| ddH_2_O | 34.5 |
| E.coli DNA polymerase I | 1 |
| E.coliRNase H/E.coli DNA Ligase Mix | 1 |

1. Incubate the samplesat 16℃for 2h and 70℃for 10 min, then add 2ul T4 DNA polymerase and incubate at 37℃for 10min.
2. Extract the double-strand DNA from reaction mixture by Agencourt RNAclean XP magnetic beads as the user’s manual. At last, add 24ul DEPC treated H_2_O to elute DNA.

**In-vitro transcription RNA synthesis and purification** (RiboMAX^TM^ Large Scale RNA Production Systems, Promega)

The T7-IlluminaA-oligod(T)primer contains a sequence of T7 promoter, so we can use the RiboMAX^TM^ System to produce large quantities of RNA. The amount of RNA can be amplified to 10-20 fold.

1. Prepare the following reagents in an autoclaved nuclease–free tube at room temperature, mix thoroughly and briefly microcentrifugate.

| **Reagents** | **Volume(μl)** |
| --- | --- |
| Above Double-strand DNA solution | 24 |
| 5×T7 Transcription buffer | 12 |
| RNTPs (25MmATP,CTP,GTP,UTP) | 18 |
| Enzyme Mix | 6 |

1. Incubate the samples at 37℃for 4h then add 1ul RQ1 RNase-free DNase (1U/ul) and incubate at 37℃for 15min.
2. Extract the in-vitro transcription RNA from reaction mixture by Agencourt RNAclean XP magnetic beads.(Add 25ul DEPC H_2_O to elute RNA)

**Second reverse-transcription cDNA synthesis and purification**

1. Prepare the following reagents in an autoclaved nuclease-free tube on ice, mix thoroughly and briefly microcentrifugate. Incubate it at 65℃ for 5 minutes, then put it on ice.

| **Reagents** | **Volume(μl)** |
| --- | --- |
| IlluminaB-Random primer (1ug/ul) | 1 |
| RNase inhibitor | 0.2 |
| In-vitro transcription RNA (~1ug) | Up to 10.6 |

1. Prepare the following reagents in an autoclaved nuclease–free tube on ice.

| **Reagents** | **Volume(μl)** |
| --- | --- |
| 5×First strand buffer (Invitrogen) | 4 |
| 0.1M DTT (Invitrogen) | 2 |
| dNTP (10mM, Invitrogen) | 1 |
| RNase inhibitor | 0.4 |
| T4 gene 32 (10mg/ml, NEB) | 1 |
| SuperScriptIII (Invitrogen) | 1 |

1. Mix the contents of the two above tubes by gently pipetting several times. Incubate the samples at25℃for 5min, 50℃for 60min and then 70℃for 15min.
2. Extract the cDNA from reaction mixture by Agencourt RNAclean XP magnetic beads.(Add 40ul DEPC treated H_2_O to elute cDNA)

**PCR amplification**

1. Prepare the following reagents in an autoclaved nuclease–free tube on ice, mix thoroughly and briefly microcentrifugate;

| **Reagents** | **Volume(μl)** |
| --- | --- |
| cDNA | 10 |
| 10 X High Fidelity PCR Buffer(Invitrogen) | 5 |
| dNTP Mixture(10mM each)(Invitrogen) | 0.3 |
| 25mM MgSO4(Invitrogen) | 4 |
| Illumina-PrimerA (10μM) | 0.45 |
| Illumina-Barcode-PrimerB (10μM) | 0.45 |
| Platinum® Taq High Fidelity(Invitrogen) | 1 |
| Autoclaved, distilled water | Up to 50 |

1. Perform PCR amplification as the following:

| 94℃  94℃ | 3min |  |
| --- | --- | --- |
| 94℃ | 10s  15cycles |  |
| 60℃ | 20s |  |
| 68℃ | 30s |  |
| 68℃ | 10min |  |
| Cooling to 12 ℃ | |  |

**library size selection with Agencourt Ampure XP magnetic beads**

After PCR with sequencing primers, specific size of fragments between 250bp and 700bp was selected with Agencourt Ampure XP magnetic beads. 0.6V beads was first added into PCR product to remove the DNA fragments larger than 700bp and then 0.2V beads was added to remove DNA fragments smaller than 250bp.

1. Add 0.6V Bead Solution to Reaction Solution Mix by pipetting 15 times, incubate at room temperature for 5min and on DynaMag (Invitrogen) for 5min.
2. Transfer the supernatant to a new tube and add 0.2V Bead Solution(relative to Reaction Solution) ,incubate at room temperature for 5min and on DynaMag for 5min.
3. Discard supernatant and wash beads with freshly prepared 70% ethyl alcohol 3 times,1min each time.
4. Air dry for 5min and elute with 20ul H_2_O.
